# Supplementary figures and images for: Spatial ecology of Haemophilus and Aggregatibacter in the human oral cavity
Source: Microbiol Spectr. 2024 Mar 15;12(4):e04017-23. doi: 10.1128/spectrum.04017-23 (PMC10986600; doi:10.1128/spectrum.04017-23)

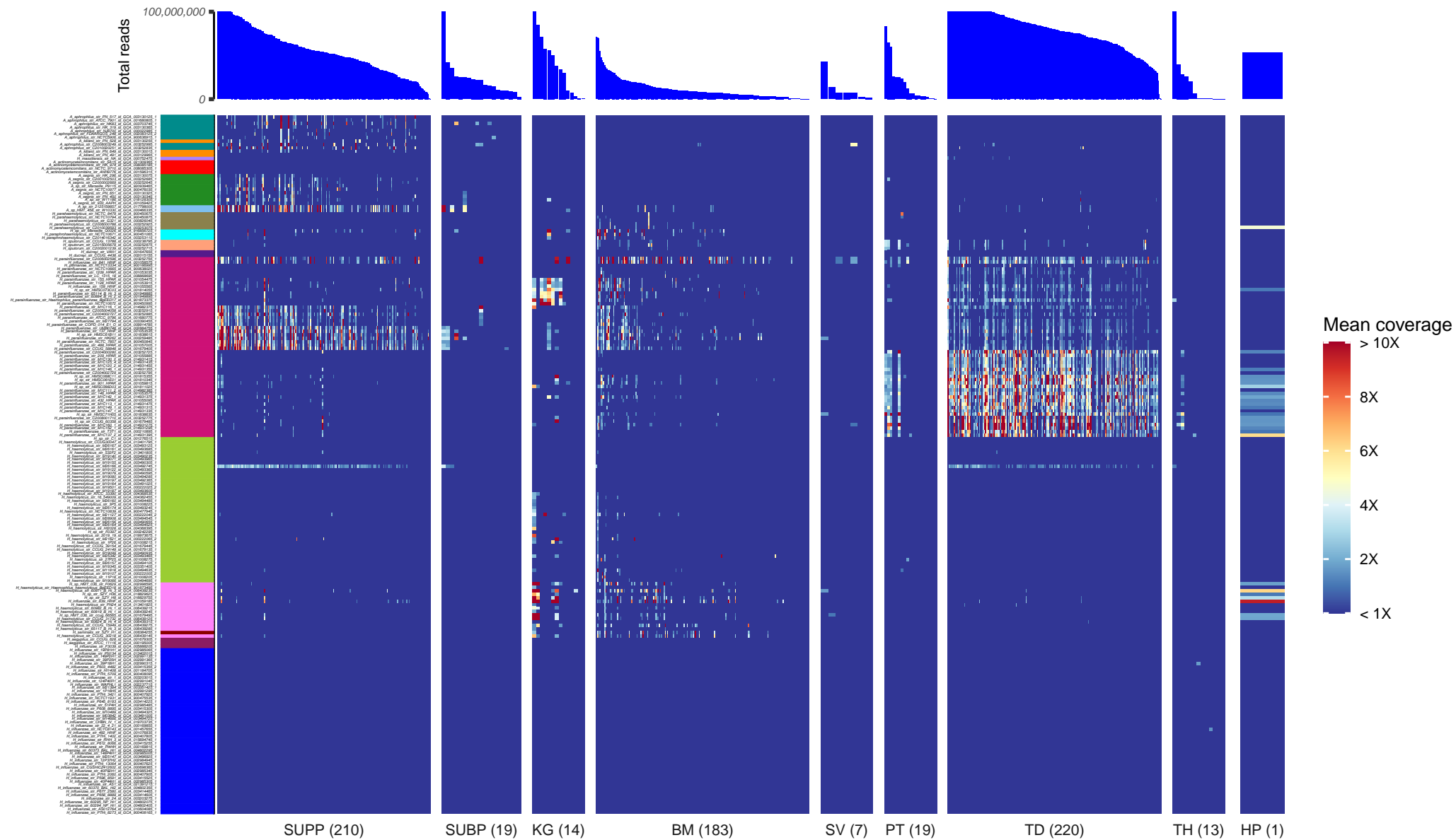

Supplement: Fig. S1 — Genome mean coverage plot. [file spectrum.04017-23-s0001.pdf]

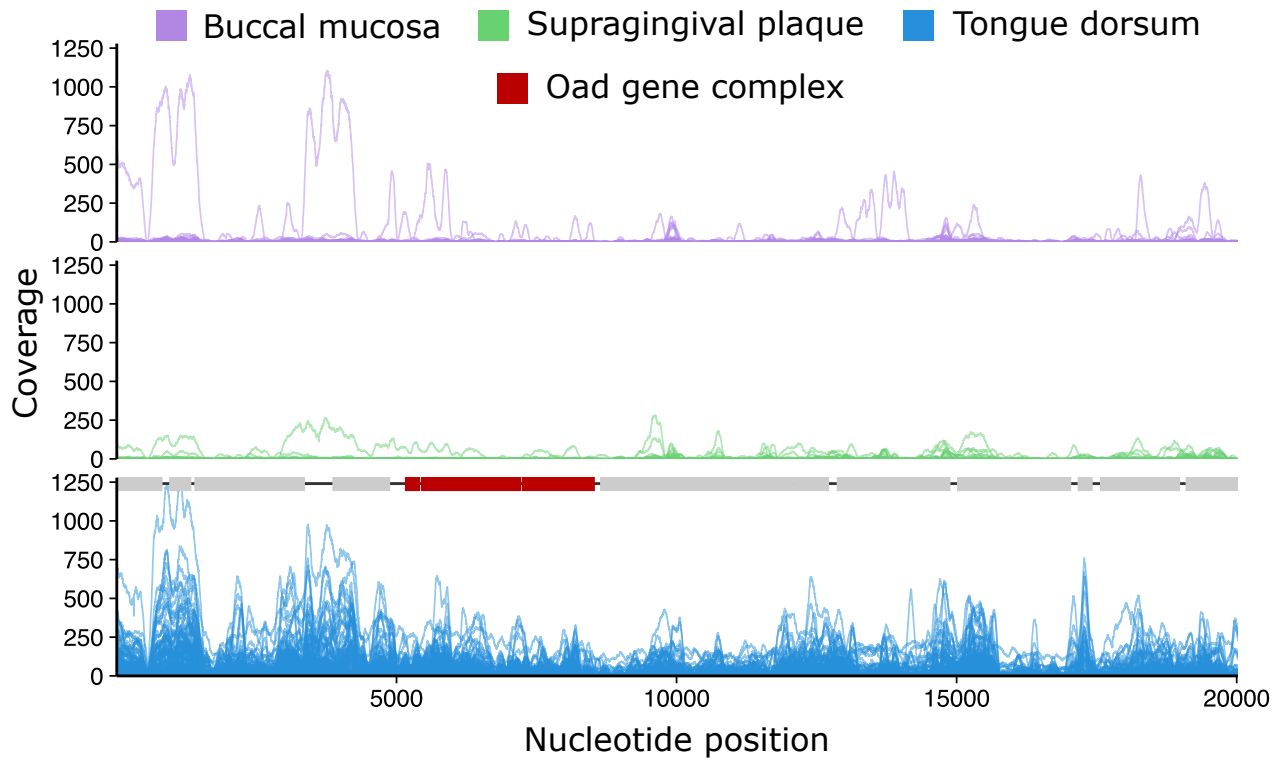

Supplement: Fig. S8 — Oad gene operon nucleotide level coverage plot. [file spectrum.04017-23-s0008.pdf]
