# Supplementary material for: Spatial ecology of Haemophilus and Aggregatibacter in the human oral cavity
Source: Microbiol Spectr. 2024 Mar 15;12(4):e04017-23. doi: 10.1128/spectrum.04017-23 (PMC10986600; doi:10.1128/spectrum.04017-23)
Supplement: Fig. S2 — Supragingival plaque ternary plot of relative abundance between three dominant plaque species. [file spectrum.04017-23-s0002.pdf]

(a)

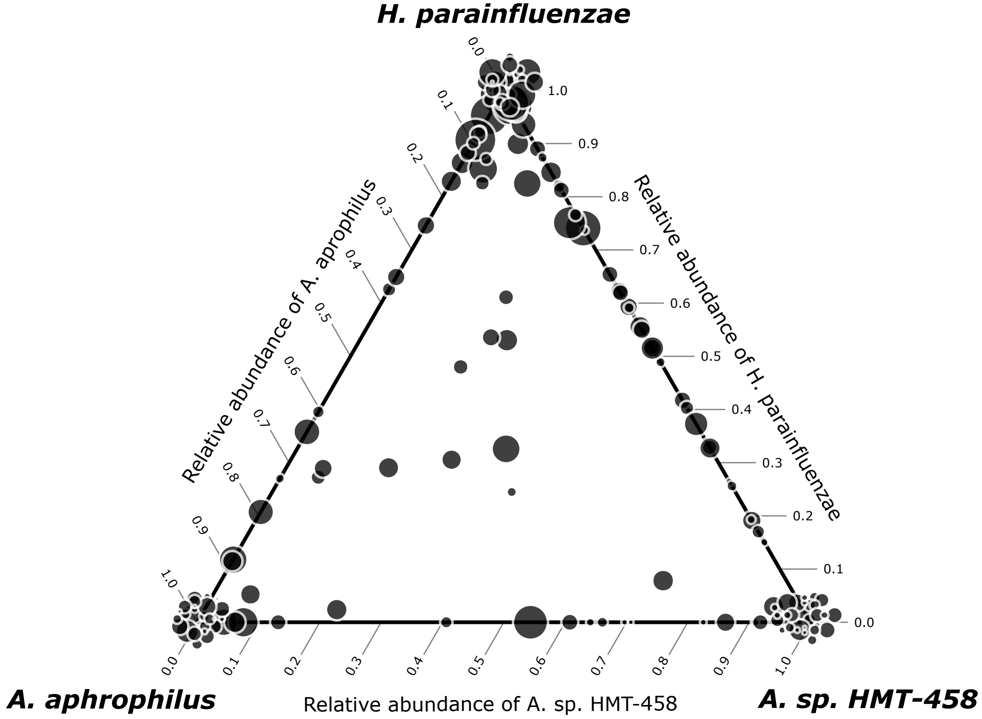


| Total | Total corner (fraction) | H. para corner (fraction) | A. aphro corner (fraction) | A. HMT-458 corner (fraction) | Total edge (fraction) | H.para - A.aphro edge (fraction) | A. aphro - A. HMT-458 edge (fraction) | H. para - A. HMT-458 edge (fraction) | Interior (fraction) |
|---|---|---|---|---|---|---|---|---|---|
| 168 | 97 (0.578) | 36 (0.214) | 29 (0.173) | 32 (0.190) | 52 (0.310) | 12 (0.171) | 10 (0.060) | 30 (0.179) | 19 (0.113) |

(b)

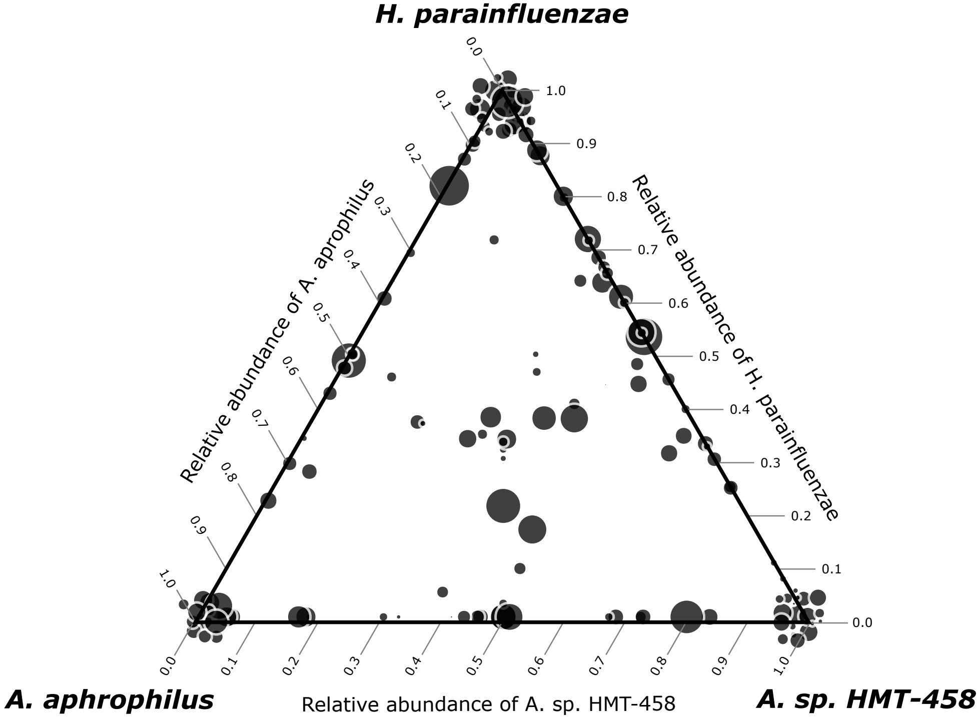


| Total | Total corner (fraction) | H. para corner (fraction) | A. aphro corner (fraction) | A. HMT-458 corner (fraction) | Total edge (fraction) | H.para - A.aphro edge (fraction) | A. aphro - A. HMT-458 edge (fraction) | H. para - A. HMT-458 edge (fraction) | Interior (fraction) |
|---|---|---|---|---|---|---|---|---|---|
| 168 | 63 (0.375) | 27 (0.161) | 15 (0.089) | 20 (0.119) | 57 (0.339) | 14 (0.083) | 18 (0.107) | 25 (0.149) | 49 (0.292) |
