# Supplementary material for: Spatial ecology of Haemophilus and Aggregatibacter in the human oral cavity
Source: Microbiol Spectr. 2024 Mar 15;12(4):e04017-23. doi: 10.1128/spectrum.04017-23 (PMC10986600; doi:10.1128/spectrum.04017-23)
Supplement: Fig. S7 — BIOA gene, genome and species coverage plot. [file spectrum.04017-23-s0007.pdf]

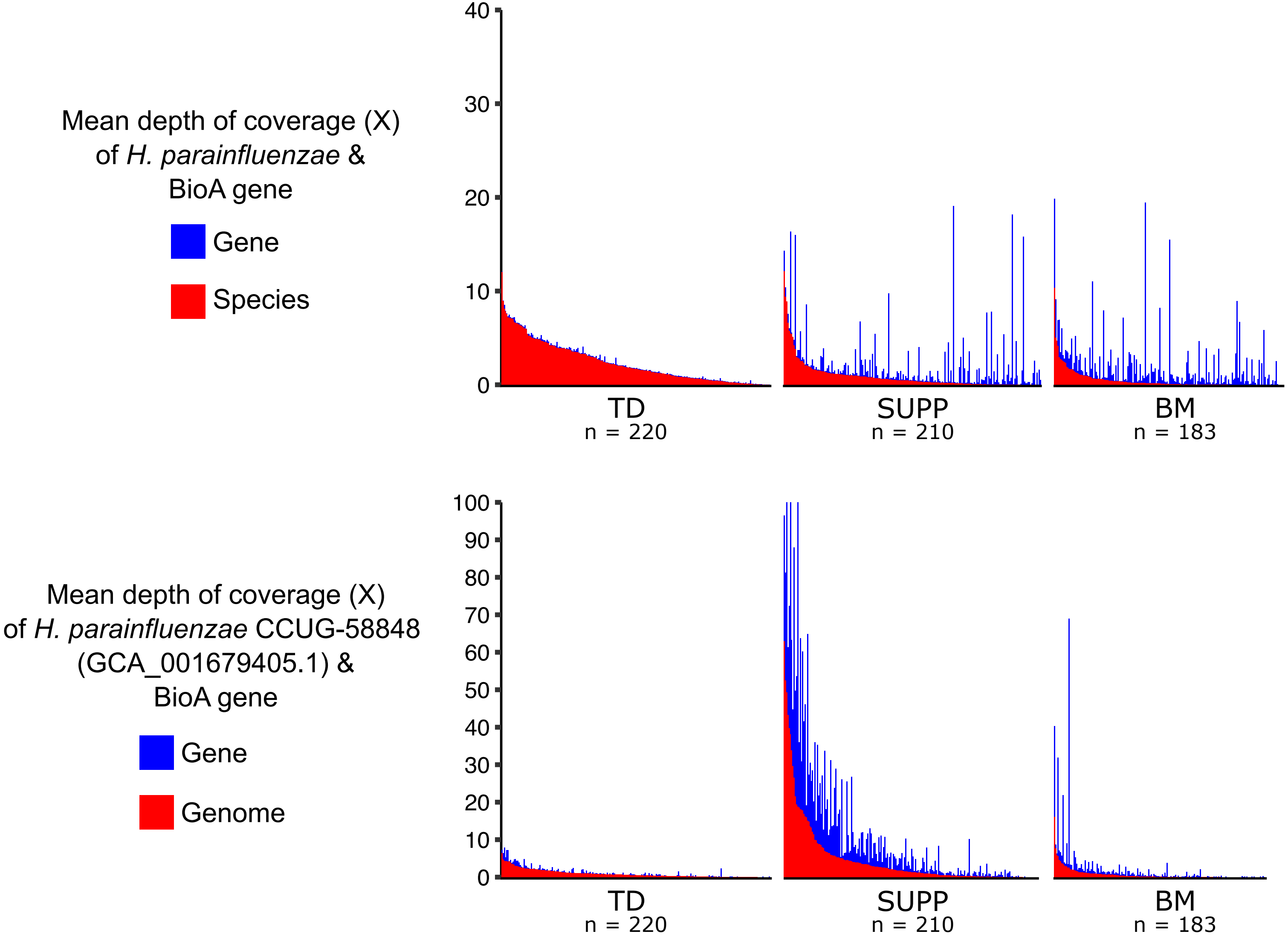
Mean depth of coverage (X)
of H. parainfluenzae &
BioA gene
Gene
Species
40
30
20
10
0
TD
n = 220
SUPP
n = 210
BM
n = 183
Mean depth of coverage (X)
of H. parainfluenzae CCUG-58848
(GCA_001679405.1) &
BioA gene
Gene
Genome
100
90
80
70
60
50
40
30
20
10
0
TD
n = 220
SUPP
n = 210
BM
n = 183
