# Supplementary material for: Spatial ecology of Haemophilus and Aggregatibacter in the human oral cavity
Source: Microbiol Spectr. 2024 Mar 15;12(4):e04017-23. doi: 10.1128/spectrum.04017-23 (PMC10986600; doi:10.1128/spectrum.04017-23)
Supplement: Supplemental material — Supplemental text and figure legends. [file spectrum.04017-23-s0009.docx]

SUPPLEMENTAL MATERIALS

Spatial ecology of *Haemophilus and Aggregatibacter* in the human oral cavity

Jonathan J. Giacomini^1,4^, Julian Torres-Morales^1^, Jonathan Tang^1^, Floyd E. Dewhirst^1,2^, Gary G. Borisy^1^, Jessica L. Mark Welch^1,3^

^1^The Forsyth Institute, Cambridge, MA 02142

^2^Harvard School of Dental Medicine, Boston MA 02115, USA.

^3^Marine Biological Laboratory, Woods Hole, MA 02543, USA

**Supplemental Text:**

***Permutation Test for three-way reciprocal relationship***

To determine the validity of the three-way reciprical relationship between *H. parainfluenzae*, *A*. sp. HMT-458 and *A. aphrophilus*, we used a permutation test to assess the statistical significance of differences between the observed data set and a set of simulated data sets. First, we generated simulated data sets by shuffling the relative abundance values for each taxa (*H. parainfluenzae*, *A.* sp. HMT-458 and *A. aphrophilus*) and sample. We then determined location of each sample in the ternary plot based on predefined rules, which was then used to calculate frequencies of each location type in the ternary plot for both the observed and simulated data sets. The rules were as follows: a sample was assigned to a taxon’s “corner” if the relative abundance of the taxon was greater than or equal to 90%; a sample was assigned to a shared “edge” between two taxa if both taxa had a relative abudance greater than 0% and less than 90%, and the third taxon had a relative abundance of 0%; all remaining samples were assigned to the “interior”. A Chi-square test of independence was then used to evaluate the association between the location and data set type. This process was repeated 1000 times and the final p-value was computed as the mean of the p-values from all chi-square tests. A p-value below 0.05 indicated that the observed data set was significantly different from the simulated data sets. The permutation test provides a rigorous and data-driven approach to assess the significance of the observed bubble locations in the ternary plot, helping determine whether the locations are likely driven by an association between the species' relative abundances or are simply due to random chance.

**Supplemental Figures:**

**Figure S1:** Heatmap shows the whole genome mean depth of coverage (Q2Q3) of *Haemophilus* and *Aggregatibacter* reference genomes (n = 202) across 9 major oral sites: supragingival plaque (SUPP), subgingival plaque (SUBP), keratinized gingiva (KG), buccal mucosa (BM), saliva (SV), palatine tonsil (PT), tongue dorsum (TD), throat (TH) and hard palate (HP). Mean depth of coverage across nucleotide positions in the 2nd and 3rd quartiles (the Q2Q3 interquartile range) was calculated after nucleotides were ranked by their depth of coverage. Rows and columns correspond to individual genomes and Human Microbiome Project metagenomic samples, respectively. Samples are ordered descending from left to right within oral sites based on the total number of reads per sample after QC filtering, which is depicted in the bar chart above each oral site column. Reference genomes are ordered and colors next to each reference genome ID is according to the pangenome (see Figure 3). Sample sizes indicated in paratheses for each oral site. For clearer visualization we inflated the width of oral sites with small sample sizes. Subject sex is indicated by F = female and M = male.

**Figure S2**: The mapping of whole-genome sequence data to the *Haemophilus* and *Aggregatibacter* pangenome reveals a reciprocal relationship between three abundant species in supragingival plaque. To illustrate this relationship, we present ternary plots showcasing the relative abundance of *H. parainfluenzae*, *A. aphrophilus*, and *A*. sp. HMT-458 in metagenomes obtained from supragingival plaque through whole-genome shotgun sequencing. Panel (a) displays the relative abundance of each of the three taxa as bubbles representing individual samples (n = 168) wherein at least one of the three taxa was present. Each bubble corresponds to a sample, and the size of the bubble indicates the number of reads that were mapped to the pangenome. In panel (b), bubbles represent an example simulated data set generated by randomly shuffling the relative abundance values of the samples. Once again, the bubble size corresponds to the number of reads mapped to the pangenome. Bubbles in which one species had 100% relative abundance were manually jittered for visualization. The tables below each panel provide summaries of bubble counts for each species based on their occurrence in different areas: the corners (where the species' relative abundance is greater than 90%), the edges (where one of the three species' relative abundance is 0%), and the interior (where all three species have relative abundances between 0% and 90%). The numbers in parentheses represent bubble counts as fractions of the total number of bubbles. This panel offers insights into the dynamics of these species over time and provides valuable information on their temporal variation. By presenting this data using ternary plots, we aim to provide a visually intuitive representation of the complex relationships and dynamics between *H. parainfluenzae*, *A. aphrophilus*, and *A*. sp. HMT-458 in supragingival plaque.

**Figure S3:** Mean depth of coverage for the Oad-A1 gene across three prevalent oral sites (TD = tongue dorsum, SUPP = supragingival plaque, and BM = buccal mucosa). In the upper panel, red bars represent average species coverage of *H. parainfluenzae* per sample, calculated by summing Q2Q3 mean depths of coverage from individual *H. parainfluenzae* reference genomes and dividing by the number of genomes (n = 54). Blue bars depict the mean depth of coverage of the Oad-A1 gene at the species level, calculated by summing coverage across all *H. parainfluenzae* reference genomes and dividing by the number of genomes (n = 54). Samples are ordered left to right based on species coverage. In the lower panel, red bars indicate the average coverage of a specific *H. parainfluenzae* reference genome (strain: M1C142-1) that exhibited pronounced site specialization on the tongue dorsum. Blue bars show the mean depth of coverage of the Oad-A1 gene for each sample relative to the selected reference genome. Samples are ordered left to right based on reference genome coverage. For visualization purposes, the y-axis in the lower panel is truncated at 100X coverage.

**Figure S4**: Mean depth of coverage for the OadG gene across three prevalent oral sites (TD = tongue dorsum, SUPP = supragingival plaque, and BM = buccal mucosa). In the upper panel, red bars represent average species coverage of *H. parainfluenzae* per sample, calculated by summing Q2Q3 mean depths of coverage from individual *H. parainfluenzae* reference genomes and dividing by the number of genomes (n = 54). Blue bars depict the mean depth of coverage of the OadG gene at the species level, calculated by summing coverage across all *H. parainfluenzae* reference genomes and dividing by the number of genomes (n = 54). Samples are ordered left to right based on species coverage. In the lower panel, red bars indicate the average coverage of a specific *H. parainfluenzae* reference genome (strain: M1C142-1) that exhibited pronounced site specialization on the tongue dorsum. Blue bars show the mean depth of coverage of the OadG gene for each sample relative to the selected reference genome. Samples are ordered left to right based on reference genome coverage. For visualization purposes, the y-axis in the lower panel is truncated at 100X coverage.

**Figure S5**: Mean depth of coverage for the OadB gene across three prevalent oral sites (TD = tongue dorsum, SUPP = supragingival plaque, and BM = buccal mucosa). In the upper panel, red bars represent average species coverage of *H. parainfluenzae* per sample, calculated by summing Q2Q3 mean depths of coverage from individual *H. parainfluenzae* reference genomes and dividing by the number of genomes (n = 54). Blue bars depict the mean depth of coverage of the OadB gene at the species level, calculated by summing coverage across all H. parainfluenzae reference genomes and dividing by the number of genomes (n = 54). Samples are ordered left to right based on species coverage. In the lower panel, red bars indicate the average coverage of a specific *H. parainlfuenzae* reference genome (strain: M1C142-1) that exhibited pronounced site specialization on the tongue dorsum. Blue bars show the mean depth of coverage of the OadB gene for each sample relative to the selected reference genome. Samples are ordered left to right based on reference genome coverage. For visualization purposes, the y-axis in the lower panel is truncated at 100X coverage.

**Figure S6**: Mean depth of coverage for the BioF gene across three prevalent oral sites (TD = tongue dorsum, SUPP = supragingival plaque, and BM = buccal mucosa). In the upper panel, red bars represent average species coverage of *H. parainfluenzae* per sample, calculated by summing Q2Q3 mean depths of coverage from individual *H. parainfluenzae* reference genomes and dividing by the number of genomes (n = 54). Blue bars depict the mean depth of coverage of the BioF gene at the species level, calculated by summing coverage across all H. parainfluenzae reference genomes and dividing by the number of genomes (n = 54). Samples are ordered left to right based on species coverage. In the lower panel, red bars indicate the average coverage of a specific *H. parainlfuenzae* reference genome (strain: CCUG-58848; GCA_001679405.1) that exhibited pronounced site specialization in SUPP samples. Blue bars show the mean depth of coverage of the BioF gene for each sample relative to the selected reference genome. Samples are ordered left to right based on reference genome coverage. For visualization purposes, the y-axis in the lower panel is truncated at 100X coverage.

**Figure S7**: Mean depth of coverage for the BioA gene across three prevalent oral sites (TD = tongue dorsum, SUPP = supragingival plaque, and BM = buccal mucosa). In the upper panel, red bars represent average species coverage of *H. parainfluenzae* per sample, calculated by summing Q2Q3 mean depths of coverage from individual *H. parainfluenzae* reference genomes and dividing by the number of genomes (n = 54). Blue bars depict the mean depth of coverage of the BioA gene at the species level, calculated by summing coverage across all H. parainfluenzae reference genomes and dividing by the number of genomes (n = 54). Samples are ordered left to right based on species coverage. In the lower panel, red bars indicate the average coverage of a specific *H. parainlfuenzae* reference genome (strain: CCUG-58848; GCA_001679405.1) that exhibited pronounced site specialization in SUPP samples. Blue bars show the mean depth of coverage of the BioA gene for each sample relative to the selected reference genome. Samples are ordered left to right based on reference genome coverage. For visualization purposes, the y-axis in the lower panel is truncated at 100X coverage.

**Figure S8:** Nucleotide-level coverage of the Oad gene complex (red) for a representative *H. parainfluenzae* tongue dorsum specialist reference genome (strain MC160-1; GCA_014931275.1). Colored lines represent coverage from the top 30 samples, ranked by median whole-genome coverage, for buccal mucosa (purple), supragingival plaque (green), and the tongue dorsum (blue). High coverage levels (exceeding 100X) of the Oad genes are observed in tongue dorsum samples, while consistently absent in all but one sample from buccal mucosa and supragingival plaque.
